# Transcriptional and Epigenetic Regulation of *KIAA1199* Gene Expression in Human Breast Cancer

**DOI:** 10.1371/journal.pone.0044661

**Published:** 2012-09-06

**Authors:** Cem Kuscu, Nikki Evensen, Deborah Kim, You-Jun Hu, Stanley Zucker, Jian Cao

**Affiliations:** 1 Department of Medicine/Cancer Prevention, Stony Brook University, Stony Brook, New York, United States of America; 2 Department of Pathology, Stony Brook University, Stony Brook, New York, United States of America; 3 Department of Medicine/Hematology & Oncology, Stony Brook University, Stony Brook, New York, United States of America; 4 Department of Research, Veterans Affair Medical Center, Northport, New York, United States of America; Cleveland Clinic Foundation, United States of America

## Abstract

Emerging evidence has demonstrated that upregulated expression of *KIAA1199* in human cancer bodes for poor survival. The regulatory mechanism controlling *KIAA1199* expression in cancer remains to be characterized. In the present study, we have isolated and characterized the human *KIAA1199* promoter in terms of regulation of *KIAA1199* gene expression. A 3.3 kb fragment of human genomic DNA containing the 5′-flanking sequence of the *KIAA1199* gene possesses both suppressive and activating elements. Employing a deletion mutagenesis approach, a 1.4 kb proximal region was defined as the basic *KIAA1199* promoter containing a TATA-box close to the transcription start site. A combination of 5′-primer extension study with 5′RACE DNA sequencing analysis revealed one major transcription start site that is utilized in the human *KIAA1199* gene. Bioinformatics analysis suggested that the 1.4 kb *KIAA1199* promoter contains putative activating regulatory elements, including activator protein-1(AP-1), Twist-1, and NF-κB sites. Sequential deletion and site-direct mutagenesis analysis demonstrated that the AP-1 and distal NF-κB sites are required for *KIAA1199* gene expression. Further analyses using an electrophoretic mobility-shift assay and chromatin immunoprecipitation confirmed the requirement of these *cis*- and *trans*-acting elements in controlling *KIAA1199* gene expression. Finally, we found that upregulated *KIAA1199* expression in human breast cancer specimens correlated with hypomethylation of the regulatory region. Involvement of DNA methylation in regulation of *KIAA1199* expression was recapitulated in human breast cancer cell lines. Taken together, our study unraveled the regulatory mechanisms controlling *KIAA1199* gene expression in human cancer.

## Introduction

Cancer is a major public health problem in the United States and many other parts of the world. Currently, twenty-five percent of deaths in the United States are due to cancer. In women, breast cancer has the highest incidence rate and is the second leading cause of death for women [1]. Identification of new biomarkers and a more complete understanding of the molecular mechanisms underlying breast cancer progression are crucial for early diagnosis and treatment.

*KIAA1199* is a recently identified novel gene and one of 1,087 long cDNAs (named KIAA) that encode large proteins (average length of KIAA family member is 872 amino acids) [2]. Based on a report describing genetic mutations of KIAA1199 in families with nonsyndromic hearing loss, this gene appears to be essential for normal auditory function [3].

The potential importance of KIAA1199 in cancer is just beginning to be recognized. The clinical relevance of KIAA1199 in cancer has been highlighted by recent reports of increased expression of *KIAA1199* in human gastric cancer and colorectal cancer [4], [5], [6]. Although the basic function of KIAA1199 remains unknown, an inverse correlation between the expression level of *KIAA1199* and disease stage/5-year survival rate suggests that KIAA1199 may be associated with cancer progression. In addition, high expression of *KIAA1199* in human breast cancer cell lines has also been reported [7]. However, the mechanisms that mediate upregulation of KIAA1199 in cancer are unknown. Therefore, the functional characterization of transcriptional regulatory elements of the KIAA1199 is necessary to ensure a more complete understanding of the tissue-specificity of *KIAA1199* expression and the function of the KIAA1199 protein from a pathological perspective.

The gene coding for human *KIAA1199* is located on chromosome 15 at the region of 15q25.1. KIAA1199 was found to have a G8 domain, containing eight conserved glycine residues and consisting of five β-strand pairs and one α-helix [8], and two GG domains, consisting of seven β-strands and two α-helices [9]. The domain functions of G8 and GG domains are unclear.

In controlling abnormal gene expression in cancer, epigenetic regulation is orchestrated with genetic regulation. Methylation is primarily considered as a molecular instrument for hypermethylation (over methylation) of promoters resulting in silencing of gene expression, for example tumor suppressor genes [10]. Studies investigating the hypomethylation (demethylation) of promoters resulting in upregulation of gene expression, for example oncogenes and growth-related genes, have recently gained considerable interest. Methylation often occurs in cytosine-guanine rich regions of DNA, referred to as CpG islands, which are commonly upstream of promoter regions [11]. For many years, global DNA hypomethylation was believed to play a crucial role in cancer progression by upregulating cancer-related gene expression, perhaps by freeing transcription factor binding sites in the promoter regions to facilitate access of transcription factors.

In this report, we describe the functional characterization of the human *KIAA1199* promoter as well as *cis*-acting elements and *trans*-acting factors on regulation of *KIAA1199* expression. Furthermore, epigenetic regulation of the *KIAA1199* promoter region was examined in human breast cancer specimens as well as in breast cancer cell lines. The results from this study demonstrate, for the first time, a regulatory mechanism for *KIAA1199* gene expression.

## Materials and Methods

### Ethics Statement

The protocol used for this study was approved by Stony Brook University Human Subjects Committee [207979-1] for characterization of the role of a novel protein (KIAA1199) in breast cancer metastasis.

### Cell Lines and Transient Transfections

All cell lines were purchased from ATCC (Manassas,VA). COS-1 monkey kidney epithelial, human fibrosarcoma HT1080 cell line, and MCF-7 and MDA-MB 231 human breast cancer cell lines were maintained in the DMEM (Invitrogen) containing 10% FBS. LNCaP and DU145 human prostate cancer cell lines were maintained in RPMI 1640 containing 10% FBS. Transfection of plasmid DNA into cells was achieved using polyethylenimine (Polysciences) and the transfected cells were incubated for 48 h at 37°C followed by biochemical and biological assays.

### Quantitative Real-Time PCR

RNA from cells was isolated using Qiagen RN easy Kit according to the manufacturer’s instructions. RNA was reverse transcribed to generate cDNA using Reverse Transcriptase (Bio Rad iScript cDNA Synthesis Kit). Quantitative real-time PCR was performed using BioRad iQ SYBR-Green Super Mix on a BioRad iQ5 Real Time PCR machine. Relative expression was calculated using the ΔΔCt method. HPRT-1 and GAPDH were used as internal controls.

### Data Mining

Expression of *KIAA1199* in human cancers was queried using the Oncomine database (http://www.oncomine.org). This is a publicly available database summarizing gene chip experiments across tissue types. Oncomine provides an infrastructure of data mining tools to query genes and data sets of interest as well as to meta-analyze groups of studies. Studies were included in comparison between normal tissues and corresponding cancers on expression level of *KIAA1199*.

### Non-radioactive 5′-primer Extension

Total RNA isolated from MDA-MB-231 using the Qiagen RNA Isolation kit was converted into cDNA by SuperscriptII reverse transcriptase using biotin labeled specific reverse primer (5′-biotin-GCCCTCTTACCTCTGGGTCT-3′). The biotin-labeled cDNA was loaded onto a 6% polyacrylamide gel and transferred onto a nylon membrane followed by detection using HRP-conjugated streptavidin.

### 5′ RLM-RACE

5′ RNA ligase-mediated rapid amplification of cDNA ends (5′ RLM-RACE) (First Choice RLM-RACE kit from Invitrogen) was employed to determine the transcription initiation site(s) of *KIAA1199* mRNA. This method amplifies cDNA only from full-length, capped mRNA, therefore allowing identification of the actual 5′ ends of mRNAs. Total RNA from human fibrosarcoma HT1080 cells was first treated with Calf Intestine Alkaline Phosphatase (CIP) to remove the 5′ phosphate from degraded mRNA, rRNA, and tRNA. Samples were then treated with Tobacco Acid Pyrophosphatase (TAP) to remove the cap structure of intact mRNAs, leaving a 5′ phosphate group on this mRNA subset only, followed by ligation of an RNA adapter to the decapped mRNAs. Reverse transcription and subsequent PCR amplification using gene-specific and adapter-specific primers were then performed to allow the 5′ ends of mRNA transcripts to be mapped. The gene specific outer primer and gene specific inner primer of *KIAA1199* are 5′-GTCAAACCGGTCAGAATGGATGAC-3′ and 5′- AGAGTGAGCCAGCTGATGGT-3′, respectively. The PCR products were then cloned in TA Cloning vectors and amplified. Ten clones were sequenced using T7 primer.

### Plasmid Constructs and Dual Luciferase Assay

A 3.3 kb fragment containing the *KIAA1199* 5′-flanking region was cloned from BAC clone 96012 (Invitrogen, Clone ID: 2215F6) using a PCR approach. Primers used for cloning 3.3 kb fragment and truncations of the 3.3 kb *KIAA1199* promoter were listed in Table S1. PCR reaction was performed in the presence of 0.5 M GC-rich resolution buffer (Roche) and resultant PCR products were cloned into pGL3 vector containing the firefly luciferase reporter gene. All constructs were confirmed by DNA sequencing. To examine the promoter activity, COS-1, MCF-7, and MDA-MB-231 cells were transiently transfected with the promoter constructs along with Renilla luciferase reporter gene (50∶1 ratio) using polyethylenimine. After 48 hours of transfection, firefly and Renilla luciferase activities were measured using the Promega Dual-Glo Luciferase Assay System.

### Electrophoretic Mobility Shift Assay (EMSA)

MDA-MB-231 nuclear extracts were incubated with biotinylated double-stranded oligonucleotides containing the AP-1 and NF-κB binding sites or corresponding mutations (Table S2). Where indicated, cold probe or antibody was added to the respective reactions 10 min before the addition of biotinylated probe. Binding reactions were performed for 30 min at room temperature. The samples were run on 6% non-denaturing polyacrylamide gel in Tris/borate/EDTA buffer and then transferred to a nylon membrane. Oligonucleotides were covalently attached to the membrane by UV-crosslinking. The DNA-protein complexes were detected on the membranes by the Chemiluminescence EMSA Kit (Thermo Scientific).

### Chromatin Immunoprecipitation (ChIP)

The ChIP assay was performed based on the Abcam X-ChIP (cross-linked) protocol using anti-c-Jun (AP-1) and anti-p65 (NF-κB) ChIP antibodies (Millipore), respectively. Quantitative real time PCR of the *KIAA1199* promoter containing the AP-1 and NF-κB binding sites was performed using an aliquot of sheared chromatin extracts (input) and immunoprecipitated chromatin (bound) with the specific primers (Table S3).

### DNA Bisulfite Treatment and Methylation-specific *PCR* (MSP)

DNA was isolated from cells using a DNeasy Kit (Qiagen) according to the product instructions. The isolated DNA was bisulfite treated using the EZ DNA Methylation Gold kit (Zymo Research) followed by PCR amplification. Methyl specific primers and unmethylated specific primers were designed by using the methprimer bioinformatic application (http://www.urogene.org/methprimer/rules). 5-Aza-dC treated Jurkat genomic DNA (NEB) and Sss.i treated genomic DNA were used as a negative and positive controls, respectively. Primers are listed in Table S4. PCR conditions were as follows: initial denaturation was 5 min at 95°C, followed by 30 cycles of 30 s at 94°C, 30 s a 58°C, and 30 s at 72°C, with a final extension of 72°C for 7 min.

### Laser Capture Microdissection (LCM) and Pyrosequencing

Cancer and normal epithelial cells in formalin-fixed, paraffin-embedded (FFPE) tissue sections were isolated by LCM technique using a Leica Laser Microscope. UV-energy was set to 82 and UV-Focus was set to 76 for the collection of cells. DNA was extracted from the isolated cells or cell lines followed by bisulfite treatment. Bisulfite sequencing primers (BSP) were used to amplify the first and second subregions. BSP primers and sequencing primers were designed by using Pyrosequencing™ Assay Design Software (Table S4). Pyrosequencing reaction was analyzed using Pyro Q-CpG Software.

### Gene Silencing

Small interfering oligonucleotides specific for human c-Jun and p65 or Jelly fish Green Fluorescent Protein gene were designed using Block-iT RNAi Designer (Invitrogen) for RNA interference. The annealed oligos were cloned into the RNAi-Ready pSIREN-Retro Q vector (Clontech). GFP shRNA was used as a control. A retroviral supernatant was obtained by co-transfection of a vector encoding the envelope gene (pAmphotropic) and a retroviral expression vector containing the c-Jun, p65, or GFP shRNA control into human embryonic kidney GP2-293 packaging cells (Clontech) according to the manufacturer's protocol. MDA-MB 231 and COS-1 cells were infected with the viral supernatant, and the cells were then selected with 4 µg/ml puromycin for 1–2 weeks. Silencing of gene expression was evaluated by real time RT-PCR of pooled resistant cells. The most effective stable knockdown cell lines were used for further analysis. Sequences of oligonucleotides were listed in Table S5.

### Statistical Analysis

Data is expressed as the mean-standard error of triplicates. Each experiment was repeated as least 3 times. Student’s t-test and analysis of variants (ANOVA) were used to assess differences with P<0.05 considered to be significant.

## Results

### Expression of *KIAA1199* in Human Cancers

**Figure 1:**
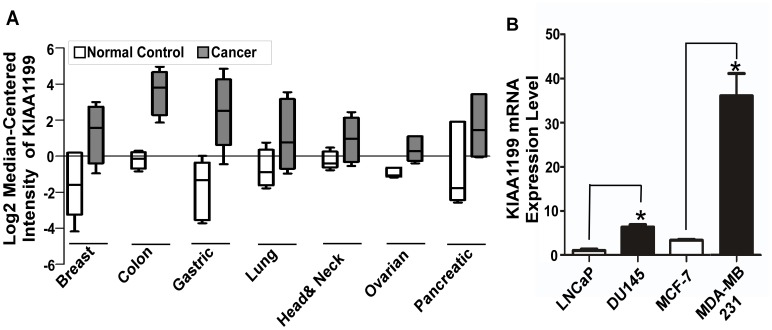
Elevated expression of *KIAA1199* in human cancers. A) By mining Oncomine and GEO databases, *KIAA1199* expression pattern in more than 40 microarray data sets shows significant alteration (P<0.01). Representative data are presented. High *KIAA1199* expression in various human cancers.1-breast cancer n:27, normal n:7, p = 6.97E-4 [41]; 2-colon cancer n:36, normal n:24, p = 1.7E-4[42]; 3-gastric cancer n:26, normal n:31, p = 3.69E-13[43]; 4-lung cancer n:45, normal n:65, p = 8.59E-9[44]; 5-head&neck cancer n:41, normal n:13, p = 2.35E-7[45]; 6-ovarian cancer n:6, normal n:4, p = 5.92E-4[46]; 7-pancreatic cancer n:11, normal n:11, p = 0.001 [47]. **B)** Expression of *KIAA1199* in human cancer cell lines: Human prostate (LNCaP and Du145), and breast cancer (MCF-7 and MDA-MB-231) cell lines were examined by real time RT-PCR using *KIAA1199* specific primers. The expression of *KIAA1199* was normalized by house-keeping genes (HPRT-1 and GAPDH). The relative levels of genes were determined using the ΔΔCt method. Each bar represents the mean ± S.E (*<0.05).

By mining DNA microarray databases Gene Expression Omnibus (NCBI) and Oncomine (Cancer Profiling Database), we observed that *KIAA1199* is significantly upregulated in various other human cancers, including human breast cancer, as compared to adjacent normal tissues examined (Fig. 1A). To determine whether these observations are recapitulated in cancer cell lines, human breast and prostate cancer cell lines were tested for expression levels of *KIAA1199*. Using real time RT-PCR, minimal expression of *KIAA1199* was found in non-invasive MCF-7 breast cancer cells and LNCaP prostate cancer cells. In contrast, high levels of *KIAA1199* mRNA were observed in invasive MDA-MB-231 breast cancer cells and DU145 prostate cancer cells (Fig. 1B). Based on these observations and the potential role of KIAA1199 in cancer progression, characterizing the regulatory mechanism of upregulated *KIAA1199* in cancer is vital to gaining a comprehensive understanding of this novel gene.

### Molecular Cloning of the Potential KIAA1199 Promoter

To identify the potential promoter region of *KIAA1199*, a bioinformatics approach employing two different promoter-prediction programs (Genomatix Model Inspector and Mc Promoter- Prediction Server at Duke University) was utilized. A PCR approach was employed using BAC clone 96012 as a template to clone the 5′-flanking region of *KIAA1199*. A 3.3 kb fragment consisting of the excess 5′-upstream of the potential promoter and the first exon of the *KIAA1199* gene was amplified and cloned into a pGL3-basic vector that lacks a promoter for the firefly luciferase reporter gene. This construct was used as a template to generate the shorter chimeric luciferase constructs used to identify the minimal promoter required for activity of *KIAA1199* in addition to *cis*-regulatory elements within the promoter.

### Determination of Transcription Start Site of KIAA1199

**Figure 2:**
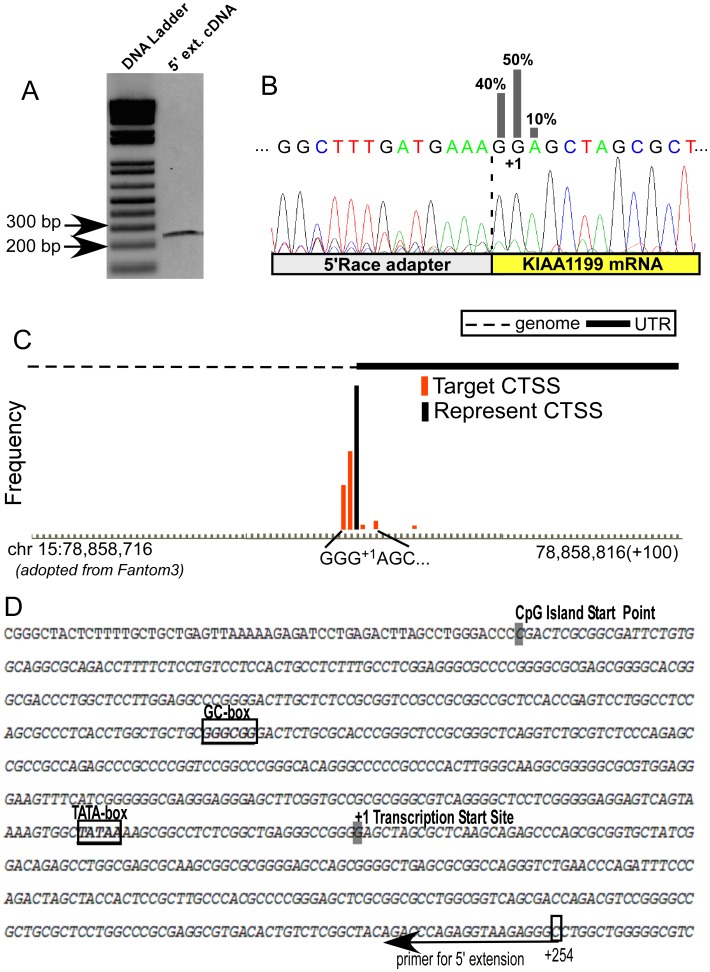
Determination of the transcription start site of the human *KIAA1199*. A) 5′ primer extension analysis: Mapping of the *KIAA1199* mRNA start site by non-radioactive 5′ primer extension analysis was performed using the reverse primer located between +254 and +234 in the first exon (shown by underlie arrow in Figure 2D). **B)** Identification of the transcription start site(s) of the *KIAA1199* mRNA: 5′ RLM-RACE was performed followed by DNA sequencing analysis. *DNA sequencing chromatogram* represents a longest transcript and percentages of the alternative transcription start sites are given. C) CAGE data analysis: One major transcription start site was identified in the CAGE analysis viewer (CTSS:Cage Transcription Start Site). **D)** Nucleotide sequence of the 5′-flanking region of the *KIAA1199*: +1 was given to the transcription start site of *KIAA1199*. According to the +1, TATA-box was identified between −31 and −27; GC-box was identified between −248 and −243. Start site of CpG island was shown with gray box in the upstream region of promoter and nucleotides in the CpG island are shown by italics.

Based on computational exon/intron junction analysis, the genomic structure of human *KIAA1199* consists of 29 exons (28 of which are coding exons) and 28 introns. The translational start site (AUG) is located within exon 2. To determine the transcription start site of *KIAA1199*, a non-radioactive 5′-primer extension approach using a biotinylated reverse primer for mRNAs isolated from MDA-MB-231 cells was performed. A single 250 bp fragment was identified, indicating the presence of a single transcription initiation site for the *KIAA1199* gene (Fig. 2A). To precisely determine the transcription initiation site(s) of *KIAA1199*, 5′ RNA ligase-mediated rapid amplification of cDNA ends (5′RLM-RACE) followed by DNA sequencing was performed using a *KIAA1199* specific primer to amply total RNA from HT1080 cells treated with Con A that enhances *KIAA1199* expression. 5′ RLM-RACE amplification of HT1080 RNA resulted in a single band with an estimated size of 375 bp. Cloning and sequencing of this PCR product revealed three distinct DNA sequences separated by one nucleotide with one major transcript start site (Fig. 2B). Based on percentage analysis, we have designated this start site as +1 (GG^+1^AGCT ). These observations are in agreement with our additional bioinformatics analysis using the Cap-Analysis Gene Expression (CAGE) database (Fig. 2C), which measures expression levels of transcription start sites by sequencing large amounts of the 5′-ends of transcripts, termed CAGE tags [12], [13].

Considering the above mentioned data and the fact that a 94 kb intron separates the promoter region and the translation start site (AUG) located within the second exon of *KIAA1199*, the transcription start site was denoted as +1 as shown in Figure 2B and 2D. Relative to the transcription start site, a canonical TATA-box in the −31/−27 region and a GC-box in the −248/−243 region were identified in the 5′-flanking region of the *KIAA1199* promoter (Fig. 2D). Our analysis suggests that the *KIAA1199* promoter is a single dominant peak promoter, which is characterized by the existence of a TATA-box and a single transcription start site [14].

### Identification of Minimal Region(s) Required for Basal Promoter Activity of KIAA1199

**Figure 3:**
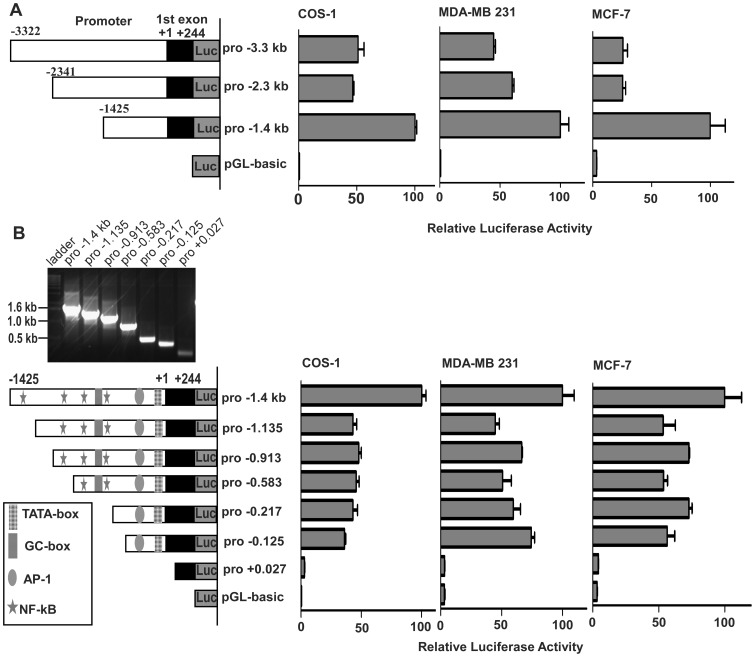
Analysis of the transcriptional activity of the *KIAA1199* promoter. **A**) *Left panel*: A schematic description of the *KIAA1199* reporter constructs containing fragments of three different lengths cloned into the pGL3-basic vector. The numbers in the names of the constructs indicate their respective lengths in nucleotides relative to the main transcription start site. *Right three panels*: Normalized firefly luciferase activity from each construct: Lysates from cells transfected with the different reporter gene constructs together with R*enilla luciferase reporter were examined for luciferase activities.* The relative promoter activities (ratio of firefly luciferase over Renilla luciferase) were compared to the activity of the pro-1.4 promoter construct (defined as arbitrary value of 100). *Error bars indicate* mean +/− S.E. **B**) *Top panel*: A ladder of PCR fragments for generating deletion mutants from the pro-1.4 promoter. *Left lower panel*: A schematic description of the pro-1.4 kb and truncations of the *KIAA1199* reporter constructs cloned in pGL3-basic vector. *Right lower three panels*: Normalized firefly luciferase activity from each construct: Lysates from cells transfected with the different reporter gene constructs together with R*enilla luciferase reporter were examined for luciferase activities. The firefly luciferase value of each sample has been normalized to its renilla luciferase value. Error bars indicate* mean +/− S.E.

To elucidate the regulatory effects of different regions within the *KIAA1199* promoter on transcriptional activity, two series of reporter constructs were generated containing fragments of the human *KIAA1199* promoter coupled to the firefly luciferase gene (Fig. 3A). We first engineered two deletion mutants by sequentially removing fragments of approximately 900 bp from the 5′-end of *pro*-3.3 (the −3.3kb construct), to generate *pro*-2.3 construct and from *pro*-2.3 to form *pro*-1.4 construct. The reporter constructs, along with a Renilla report gene that serves as a normalization control, were transfected into cells expressing endogenous *KIAA1199* (for example MDA-MB-231) (Fig. 1B) and non-detectable levels of *KIAA1199* (for example MCF-7 and COS-1 cells). The 5′-flanking region containing −1.425 kb relative to +1 site is sufficient for near maximal activity of *KIAA1199* promoter in both *KIAA1199* high and low cell lines (Fig. 3A). In contrast, expression of constructs *pro*-2.3 and *pro*-3.3 in these cells demonstrated a decrease in luciferase activity. These data suggest that activating elements are located in *pro*-1.4, whereas repressor element(s) is located in the region from −1425bp to −3322 bp. Since upregulated *KIAA1199* has been observed in human cancers, we focused on the activating elements within *pro*-1.4 to determine the minimal region required for *KIAA1199* promoter activity.

To identify minimal regions required for transcriptional activity of the *KIAA1199* promoter, we then performed an extensive mutational analysis of the −1425bp *KIAA1199* promoter (*pro*-1.4). A series of deletion mutants of *pro*-1.4 were generated and expressed in COS-1 cells followed by a Dual-Luciferase® reporter assay. Two areas were found to contribute to *KIAA1199* gene regulation, including −1425 to −1135, and −125 to +27, as demonstrated by a significant decrease in luciferase activity upon deletion of these regions (Fig. 3B). Removal of the transcription start site in construct *pro*-0.125 resulted in a significant reduction of luciferase activity to levels observed in cells expressing empty pGL3-basic vector (Fig. 3B). These observations were recapitulated in *KIAA1199*-high MDA-MB-231 cells as well as in low MCF-7 cells (Fig. 3B). Deletions from −1135 to −125 (*pro*-1.135, *pro*-0.913, *pro*-0.583, *pro*-0.217) did not result in significant changes in promoter activity, indicating a lack of critical regulatory elements within the region spanning −1135 to −125 of the *KIAA1199* promoter.

### Computational Analysis of the Putative Transcription Factor-binding Sites within the KIAA1199 Promoter

**Figure 4:**
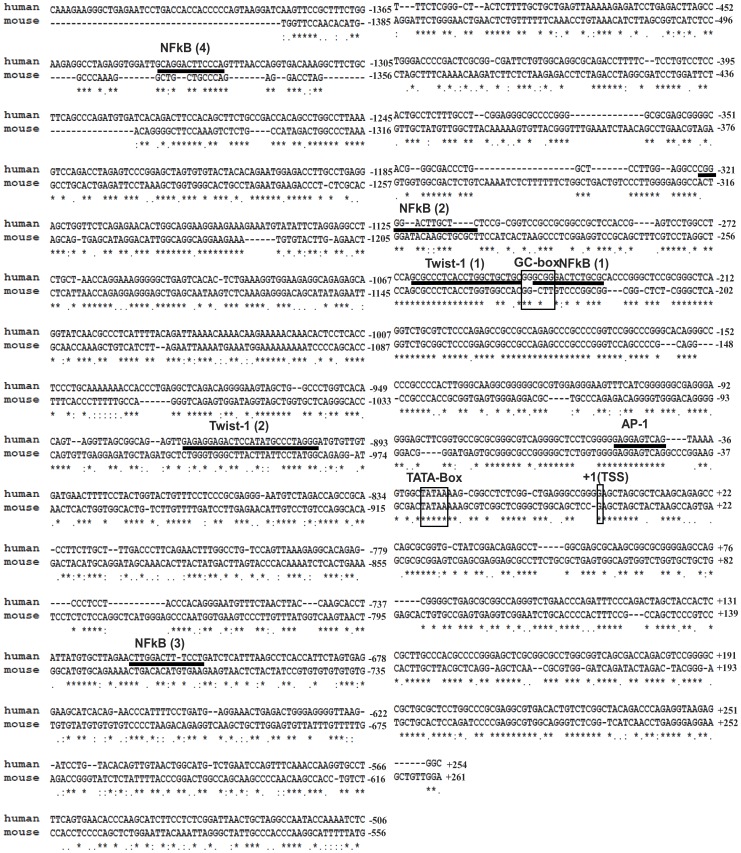
Sequence alignment of the *KIAA1199* promoter between human and mouse genomes and putative transcription factor-binding sites. Putative transcription factor-binding motifs are underlined and TATA/GC boxes and transcription start site are shown in the box. The asterisks mark the fully conserved sequences across the species.

To identify putative transcription factor-binding sites within the *KIAA1199* promoter, we employed two programs (MatInspector and Alibaba2) to predict transcription factor binding sites based on DNA sequence. This analysis revealed transcriptional elements in the *KIAA1199* promoter, including AP-1, NF-κB, and Twist-1, which are situated in the activator region (*pro-*1.4) (Fig. 4). AP-1 binding sequence (GAGT) that is located between −48 and −45 and its flanking regions on both sides are highly conserved (100%) between human and mouse. Although four putative NF-κB transcription binding sites were recognized, the first three putative NF-κB binding sites located in the proximal part of the promoter (−246/−234, −324/−312, and −704/−715), may not be involved in activation of the *KIAA1199* promoter based on the finding that deletion mutants containing these three NF-κB binding sites did not affect transcriptional activity of the *KIAA1199* promoter (Fig. 3B). In contrast, the deletion mutant encompassing the fourth putative NF-κB binding site (−1345 and −1333) lost more than 50% transcriptional activity (Fig. 3B). In addition to AP-1 and NF-κB binding sites, two putative Twist-1 binding sites (−269/−249 and −923/−903) were also identified. Deletion of the regions containing these Twist-1 putative binding sites did not notably change luciferase activity. However, since transcription factors are often conditionally expressed in certain pathological conditions, the role of AP-1, NF-κB, and Twist-1 in transcriptional activation of *KIAA1199* needs to be further examined.

### Requirement of the AP-1 Element in Activation of the KIAA1199 Promoter

**Figure 5:**
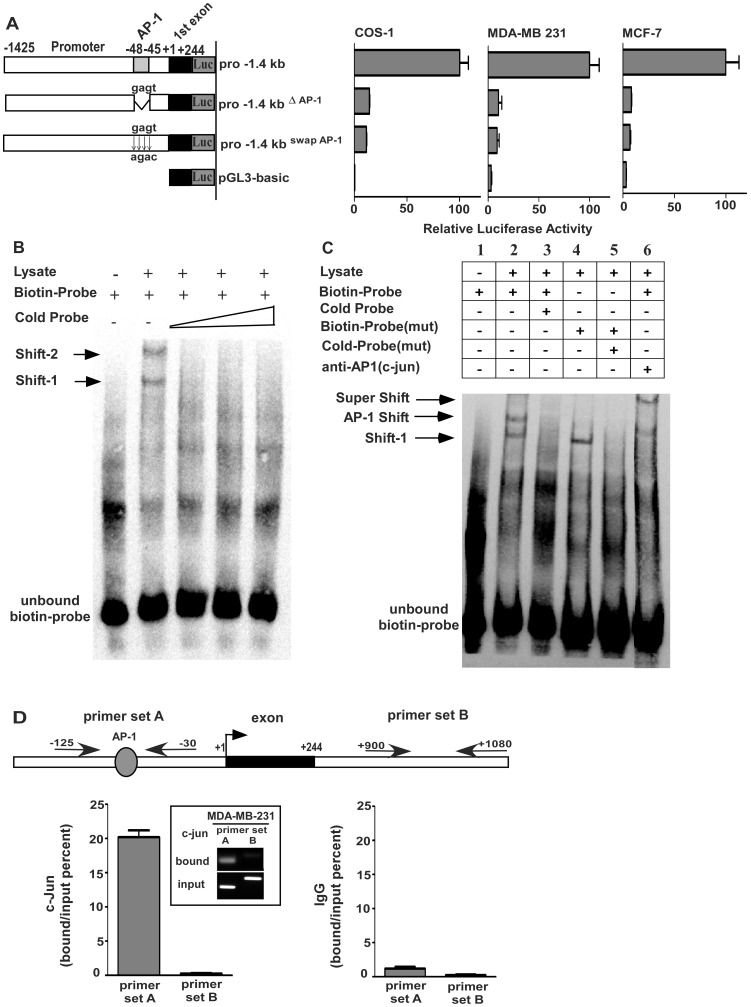
Requirement of AP-1 binding element in the *KIAA1199* promoter. **A)** A schematic diagram of mutations at the AP-1 binding site: A site-directed mutagenesis was carried out to generate either a deletion mutant by removing the AP-1 consensus sequence (GAGT) or a substitute mutation within the pro-1.4 promoter construct. The relative promoter activities of the mutations (ratio of firefly luciferase over Renilla luciferase) were then compared to the activity of the wild type pro-1.4 promoter construct (defined as arbitrary value of 100) in COS-1, MDA-MB-231, and MCF-7 cells. *Error bars indicate* mean +/− S.E. **B)** Binding of nuclear proteins to the AP-1 site in the *KIAA1199* promoter: EMSA was carried out using a biotinylated double-stranded oligonucleotide (50 bp) containing the AP-1 binding site and nuclear extracts from MDA-MB-231 cells. Where indicated, binding was competed with 50–200 fold excess amounts of unlabeled probe. DNA-protein complexes formed are indicated as Shift-1 and Shift-2. **C)** Determination of specific binding between AP1 and the *KIAA1199* promoter: EMSA was performed using a biotinylated double-stranded oligonucleotide (50 bp) containing the AP-1 binding site and nuclear extracts from MDA-MB-231 cells. Where indicated, biotinylated probe along with anti-C-Jun antibody or biotinylated probe containing mutated site of AP-1 consensus sequence were incubated with nuclear extracts from MDA-MB-231 cells. **D)** ChIP assay for analysis of association between endogenous AP-1 and the KIAA199 promoter sequence: A strong relation between AP-1 and DNA sequence was shown by 20% bound/input ratio as compared to the unrelated intron region. Normal rabbit IgG was used as a negative control. Results were calculated according to the bound/input ratio.

To examine the role of AP-1 in *KIAA1199* promoter activity, we generated additional reporter gene constructs in which the putative AP-1 binding core sequence (GAGT) was either deleted (pro-1.4^ΔAP−1^) or mutated to AGAC (pro-1.4^Swap AP−1^) by site-directed mutagenesis. The engineered mutants coupled to firefly luciferase plasmid were co-transfected with Renilla plasmid into COS-1 cells followed by a Dual-Luciferase® reporter assay. Mutations of the AP-1 site either by a deletion or substitution approach resulted in significant loss (80%) of luciferase activity (Fig. 5A). This observation was reproducible in *KIAA1199*-high (MDA-MB-231) and -low (MCF-7) cells (Fig. 5A), suggesting the requirement for AP-1 in activation of the *KIAA1199* promoter.

To determine whether there is direct binding between AP-1 transcription factor to the AP-1 binding site, an electrophorectic mobility shift assay (EMSA) was employed. Incubation of nuclear extracts from MDA-MB-231 cells with a biotinylated double-stranded oligonucleotide containing the AP-1 binding site derived from the *KIAA1199* promoter, produced two shifted bands (Shift-1 and Shift-2) (Fig. 5B). These shifted bands did not occur in the presence of additional non-biotinylated probes (cold probes). To further analyze these shifted bands, a probe containing a mutated AP-1 consensus sequence was generated and incubated with the nuclear extracts of MDA-MB-231 cells followed by EMSA. Incubation of the nuclear extract with the biotinylated-mutant probe abolished the Shift-2 band, but had no effect on the Shift-1 band, suggesting that the Shift-2 band represents a specific interaction between AP-1 and its corresponding binding sequence (Fig. 5C). An antibody supershift experiment was also performed by sequentially adding the anti-c-Jun (AP-1) antibody to the binding reaction in the nuclear extracts containing biotinylated AP-1 probe. Co-incubation of antibody against c-Jun with nuclear extracts and AP-1 probe diminished formation of the Shift-2 band and resulted in formation of a super shift band (Fig. 5C, lane 6). This is due to the formation of a ternary complex consisting of the AP-1 probe, the AP-1 transcription factor, and the anti-AP-1 antibody.

To further assess if endogenous AP-1 binds to the AP-1 consensus sequence within the *KIAA1199* promoter, a chromatin immunoprecipitation (ChIP) assay was utilized. Since the AP-1 consensus sequence is known to be recognized by a transcriptional complex composed of either homodimer of Jun family proteins or a heterodimer of Jun and Fos molecules, we employed anti-c-Jun antibody to precipitate AP-1 followed by real time RT-PCR. Cross-linked chromatin was prepared from MDA-MB-231 cells. The *KIAA1199* promoter region containing the AP-1 binding site was precipitated using either the anti-c-Jun antibody or rabbit IgG control. Two primer sets: Set A spanning the AP-1 region from −125 to −30 and Set B spanning an unrelated region in the first intron from +900 to +1080 were designed for quantitative real time PCR analysis of either anti-AP-1 or anti-IgG antibody-precipitated chromatins. As shown in Figure 5D, precipitation with anti-AP-1 antibody, but not IgG control, resulted in amplification of the region encompassing the AP-1 site. No PCR amplification of the immunoprecipitated chromatin was detected with primer Set B for the region that does not have the AP-1 consensus sequence, thus confirming the specificity of the results.

The role of AP-1 in regulation of *KIAA1199* expression was also characterized by employing both gain- and loss-of-function studies. Overexpression of cDNAs encoding c-Jun, an AP-1 subunit (Fig. S1A), resulted in a significant induction of endogenous *KIAA1199* examined by real time RT-PCR as compared to cells transfected with vector controls (Fig. S1B). Correlatively, silencing of c-Jun in MDA-MB-231 cells displayed decreased *KIAA1199* expression (Fig. S2). Furthermore, silencing c-Jun in COS-1 cells resulted in reduced promoter activity of pro-1.4kb as compared to GFP shRNA control (Fig. S3A and S3C). Together, these data indicate that the *KIAA1199* promoter contains a functional AP-1 binding site that plays a critical role in transcriptional activation of the *KIAA1199* promoter.

### Involvement of NF-κB in Transcriptional Activity of the KIAA1199 Promoter

**Figure 6:**
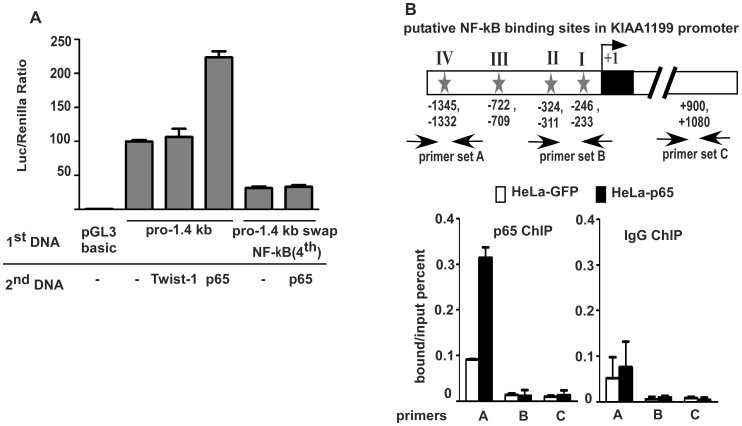
Characterization of NF-κB as a regulatory element in distal part of promoter. **A)** Luciferase reporter gene assay for the effect of NF-κB and Twist-1 on the activity of *KIAA1199* promoter: Lysates of COS-1 cells transfected with cDNAs as indicated were examined by Dual Luciferase activity assay. Wild type and substitute mutation (pro-1.4 kb swap NF-κB^4th^) at distal region of NF-κB binding site were used. **B)** ChIP assay for identification of the interaction between NF-κB (p65) and *KIAA1199* promoter. Top panel: A schematic diagram of four putative NF-κB binding sites relative to +1 site. Primer sets A, B, and C were designed for NF-κB binding site IV and I + II, and an area of the first *KIAA1199* intron, respectively. Middle and low panels: ChIP PCR in HeLa cells transfected with GFP control and P65 cDNAs. Anti-p-65 antibody and normal IgG (control) were used for immunoprecipitation. Results were calculated according to the bound/input ratio.

NF-κB and Twist-1 transcription factors have been demonstrated to play important roles in epithelial-to-mesenchymal transition (EMT), an early and critical step for cancer invasion and metastasis [15], [16]. To examine whether the putative NF-κB and Twist-1 sites are required for *KIAA1199* transcription activity, we first tested if overexpression of NF-κB (p65 cDNA) or Twist-1 in cells results in enhanced *KIAA1199* promoter activity. COS-1 cells were transiently co-transfected with *KIAA1199* promoter pro-1.4 kb along with either NF-κB p65, a subunit of the NF-κB transcription complex, or Twist-1 cDNAs followed by a luciferase activity assay. In agreement with our observation derived from the study with deletion mutants (Fig. 3B), overexpression of NF-κB p-65, but not Twist-1 cDNAs, significantly enhanced *KIAA1199* transcription activity as compared to vector control with the *pro*-1.4 kb promoter construct (Fig. 6A). Silencing of NF-κB (p65) in COS-1 cells resulted in reduced promoter activity of pro-1.4 kb as compared to GFP shRNA control (Fig. S3B and S3C). In addition, the effect of NF-κB, but not Twist-1, in regulation of *KIAA1199* expression was confirmed by gain-of-function study (Fig. S1). Since these data suggested that Twist-1 was not involved in induction of *KIAA1199*, only NF-κB was further examined to determine which putative site(s) is involved in *KIAA1199* promoter activity. To this end, a site-direct mutagenesis approach was employed to substitute key consensus nucleotides within the deduced binding sites based on predicted high Consensus Index score (Ci-value) as indicated by MatInspector. The mutant plasmids were co-transfected into MDA-MB-231 cells along with Renilla plasmid followed by a Dual-Luciferase reporter assay. No significant change in luciferase activity was observed upon mutation of the three NF-κB sites in the proximal region of *KIAA1199* promoter relevant to +1 (data not shown). However, substitution of the distal NF-κB binding site reduced the promoter activity by 75% compared to the full length 1.4 kb promoter (Fig. 6A). In addition, overexpression of NF-κB p65 cDNA in cells expressing pro-1.4 Swap NF-κB (4^th^) failed to enhance *KIAA1199* promoter activity (Fig. 6A), suggesting the fourth NF-κB binding site may interact with NF-κB.

To further confirm the association of NF-κB with its binding consensus sequence in the *KIAA1199* distal promoter region, we performed ChIP analysis in HeLa cells transfected with GFP or NF-κB p65 cDNAs. Cross-linked chromatin from the transfected HeLa cells was immunoprecipitated using anti- NF-κB p65 and anti-IgG (control) antibodies. Three primer pairs for *KIAA1199* were designed for real time PCR as illustrated in Figure 6B: Set A covering the fourth NF-κB binding site, Set B covering the first and second NF-κB binding sites, and Set C covering an unrelated region in the first intron region. As expected, a three-fold increase in the bound over input ratio was seen in NF-κB p65 transfected cells as compared to GFP control (Fig. 6B). No difference was noted using primer Set B and C or using normal IgG control. Taken together, these data confirm that only the fourth NF-κB binding site within the distal regulatory region of the *KIAA1199* promoter is accessible to NF-κB.

### Identification of CpG Island in KIAA1199 Regulatory Region

**Figure 7:**
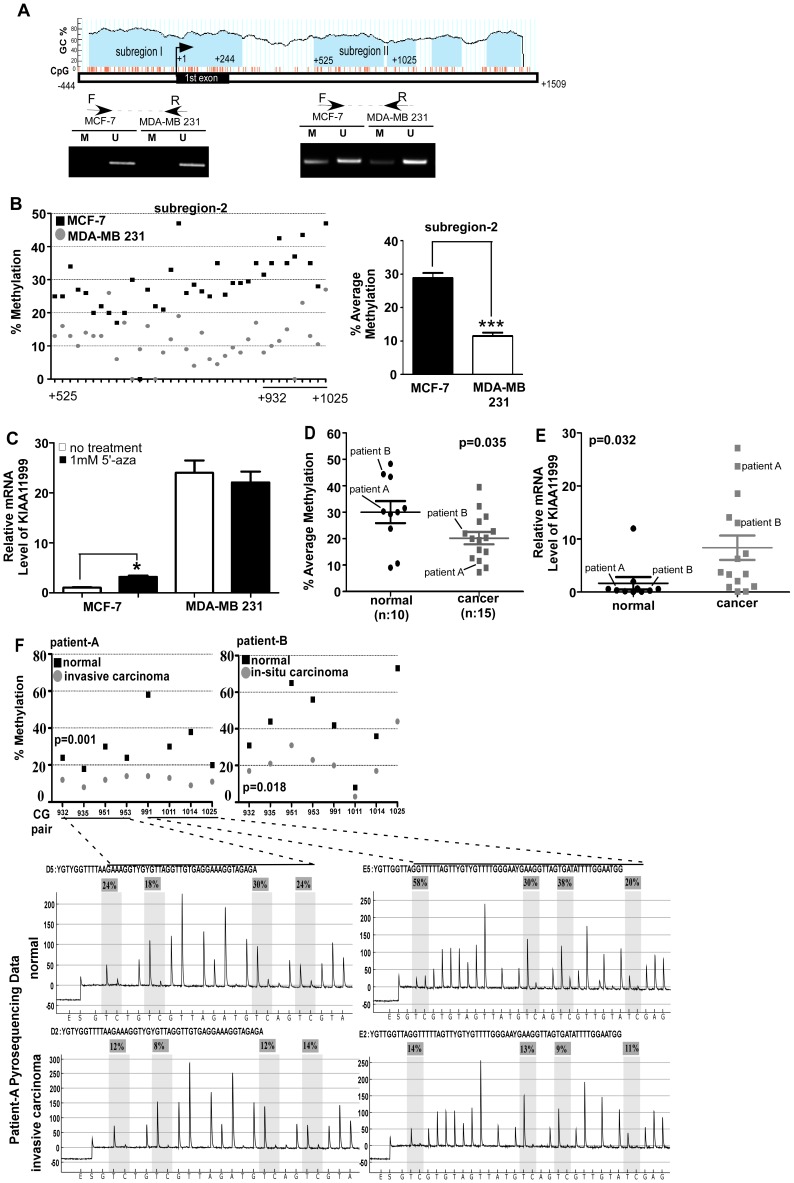
Suppression of *KIAA1199* expression in less invasive cancer cells by DNA methylation. **A)** Bioinformatics analysis of the CpG island of *KIAA1199*: Two major subregions within the CpG island were examined by MSP method for MCF-7 and MDA-MB-231 cells. F: forward primers and R: reverse primers. **B)** Pyrosequencing analysis for quantitation of methylation of cytosine residues in the second subregion of the CpG island between MCF-7 and MDA-MB-231 cells: The percentage of methylation for 36 CG pairs between +525 and +1059 was shown individually (Left panel). Average methylation rate was calculated for MCF-7 and MDA-MB-231 cells (p<0.001) (Right panel). **C)** Effect of 4 days treatment with 5′-azaon *KIAA1199* expression: Real time RT PCR was performed in MCF-7 and MDA-MB-231 cells treated with 5′-aza using *KIAA1199* specific primers. Housekeeping genes were used to normalize the gene expression. **D)** Methylation status of *KIAA1199* in human breast cancer specimens: Human invasive breast cancer cells as well as normal breast epithelial cells were harvested by LCM and pyrosequencing was performed using bisulfite-treated DNA. The average methylation level in normal cells was found higher than in cancer cells. A pair of normal breast epithelial cells and breast cancer cells from patients A and B was labeled. n: case number. **E)** *KIAA1199* expression in human breast cancer specimens: Total RNA from micro-dissected human breast cancer cells as well as normal breast epithelial cells was examined by real time RT-PCR using *KIAA1199* specific primers. Housekeeping genes were used to normalize the gene expression. A pair of normal breast epithelial cells and breast cancer cells from patients A and B was labeled. n: case number. **F)** Hypomethylation of *KIAA1199* in invasive human breast cancer specimens: Bisulfite-treated DNA from paired normal and cancer cells of breast cancer specimens harvested by laser microdissection technique was examined by a pyrosequencing approach. Representative methylation profile between the +932 and +1025 was shown for the benign and invasive cells of single patients.

Accessibility of transcription factors to their corresponding binding sequences in a promoter area is dependent on the methylation status of a promoter. Since KIAA1199 has been found to be highly expressed in human cancers as compared to normal tissues, we examined whether methylation of the *KIAA1199* promoter is a possible mechanism controlling its expression in human cancer tissues and in aggressive cancer cell lines. To determine whether methylation occurs in the *KIAA1199* regulatory region, we first searched for the presence of predicted CpG islands in the portal region surrounding the transcription start site. Employing a CpG island search program (UCSC Genome Browser), a potential 1.9 kb-long CpG island between −444 bp and +1509 bp (relevant to +1) was identified. The CpG island of *KIAA1199* contains a high GC content (66.7%) with an observed CpG/expected CpG ratio (Obs_CpG_/Exp_CpG_) of 0.729, as calculated by CpG island Searcher (http://cpgislands.usc.edu/). Given that the defining criteria for a CpG island requires a minimum 200-bp stretch of DNA with a C+G content of 50% and an Obs_CpG_/Exp_CpG_ in excess of 0.6 [17], the *KIAA1199* promoter region may contain a functional CpG island. Further analysis showed that there are two sub-regions in the CpG island based on their relatively higher GC content with respect to the rest of the island. The first sub-region was identified between −444 and +280 mainly in the proximal part, and the second one was identified between +525 and +1059 in the first intron (Fig. 7A).

Since the *KIAA1199* promoter contains a TATA box and a report suggested that CpG islands often do not present in the TATA-containing promoter [14], we examined whether the deduced CpG island within the *KIAA1199* promoter is methylated in minimal *KIAA1199* expressing cells by employing methylation-specific PCR (MSP). MSP distinguishes between unmethylated and methylated CpG islands by using two sets of primers that amplify either unmethylated or methylated sequences after bisulfite treatment, which specifically converts unmethylated cytosines to uracils. DNAs from MCF-7 (low expression of *KIAA1199*) and MDA-MB-231 (high expression of *KIAA1199*) cells treated with bisulfite were amplified by PCR using two sets of primers that were confirmed by employing artificial methylated and unmethylated human genomic DNAs (Fig. S4). In the CpG island region one, amplification only occurred using unmethylated primers in both cell lines, suggesting no methylation at cytosine residues in this region. In contrast, PCR products were amplified using both methylated primers and unmethylated primers in both MCF-7 cells and MDA-MB-231 cells. To quantitate individual methylated CpG sites, we performed pyrosequencing for both regions in MCF-7 and MDA-MB-231 cells (Fig. 7B). In agreement with our methylated PCR data (Fig. 7A), no methylation was observed in the first sub-region of the CpG island that overlaps with the proximal part of promoter encompassing the AP-1 binding consensus sequence (data not shown). Notably, the fourth NF-κB binding site is 700 bp away from the beginning of the CpG island. Therefore, DNA methylation has no or minimal direct effect on the binding of the transcription factors AP-1 and NF-κB to the promoter of *KIAA1199*. In contrast, average methylation of the second CpG island in MCF-7 cells displayed a significantly high level as compared to MDA-MB-231 cells (p<0.001) (Fig. 7B).

### Hypomethylation of the KIAA1199 Regulatory Region in Human Breast Cancer Specimens

To determine the contribution of the methylation of *KIAA1199* to the expression of this gene, a common demethylating agent, 5′-azacytidine (5′-aza), was used. *KIAA1199* expression in MCF-7 and MDA-MB-231 cells treated with 5′-aza was evaluated by a real time RT-PCR approach using specific primers as shown in Figure 1. MCF-7 cells treated with 5′-aza resulted in a three-fold increase of *KIAA1199* expression (p = 0.014), while no statistical significance was seen in MDA-MB-231 cells (Fig. 7C), suggesting the possible role of methylation in regulating *KIAA1199* expression.

To determine the methylation status of the *KIAA1199* regulatory region in human breast cancers, we investigated the methylation level of CpG sites within the *KIAA1199* regulatory region between +932 and +1025 in 15 human breast cancer tissues and 10 normal breast tissues that were collected from corresponding breast cancer patients. To precisely determine methylation status of breast cancer tissues, a laser-capture microdissection (LCM) method was employed to collect the malignant cancer cells as well as benign epithelial cells from the same breast cancer specimen as well as normal breast epithelial. Real time RT-PCR demonstrated that *KIAA1199* mRNA is highly upregulated in human breast cancers as compared to normal tissues (manuscript submitted). Bisulfite-treated DNA was isolated from microdissected cells (Fig. S5) followed by pyrosequencing to quantitatively analyze the methylation status of eight CpG sites in malignant breast cancer cells and normal breast epithelial cells. Pyrosequencing analysis revealed with high reproducibility that neighboring CpG sites in breast cancer specimens showed low degrees of average methylation as compared to normal controls (p = 0.035) (Fig. 7D). Significantly, DNA methylation status was inversely correlated with *KIAA1199* expression in patient-matched pairs of normal breast epithelial cells from breast cancer cells (Fig. 7E). Representative pyrosequencing data from one patient with its invasive and normal cells are shown in Figure 7F. The prevalence of *KIAA1199* promoter demethylation in breast tumors is strongly consistent with the enhanced expression of *KIAA1199* in human breast cancer tissues and aggressive breast cancer cell lines (Figs. 1 and 7E).

## Discussion

Gene expression is tightly controlled by both genetic and epigenetic regulatory mechanisms. It is generally accepted that *cis*- and *trans*-regulatory machineries are basic requirements for gene expression and that DNA methylation regulates DNA-protein interactions that can interfere with gene transcription. The results of the experiments described in this study suggest the tight regulation of a novel cancer-related gene, *KIAA1199*, in human breast cancer. A mutagenesis study involving a series of truncated *KIAA1199* promoters was successful in identifying the basic promoter region required for *KIAA1199* expression (Fig. 3). A site-direct mutagenesis study demonstrated the existence of *cis*-acting elements, specifically AP-1 and NF-κB sites, within the *KIAA1199* basic promoter (Figs. 5 and 6). EMSA and ChIP results confirmed the requirement for *trans*-acting elements of AP-1 and NF-κB transcription factors in regulating *KIAA1199* expression. The basic promoter activity of *KIAA1199*, however, is dependent on the DNA methylation status with the CpG island located within the first intron of *KIAA11999*. Furthermore, the link between hypomethylation and upregulated *KIAA1199* in human breast cancer has been established. Thus, *KIAA1199* is coordinately regulated through genetic and epigenetic mechanisms to control its gene expression.

Although the Human Genome Project is now completed, many obscured genes, such as *KIAA1199,* remain to be characterized. The importance of this novel *KIAA1199* gene has been highlighted by recent reports showing that upregulated expression of *KIAA1199* occurs in human cancers and correlates with patient survival probability [4], [5], [6]. However, the function of KIAA1199 in cancer progression has not been previously reported. Our recent efforts in characterizing the role of KIAA1199 in cancer progression (manuscript submitted), in addition to the high expression in human cancers, strongly suggests that KIAA1199 plays an important role in cancer dissemination.

The *KIAA1199* promoter is a classic promoter with its relatively simple organization and tight regulation limited to a few structural elements such as the canonical TATA- and GC-boxes, and the AP-1 and NF-κB *cis*-binding elements. It has been demonstrated that TATA-box containing genes often contain several transcription start sites [13]. We observed one major transcription start site within the *KIAA1199* 5′-flanking region based on 5′ primer extension analysis and 5′RLM RACE experiment, which are common approaches used to determine transcription start sites [18]. Our analysis using the CAGE database, which is primarily used to locate transcription start sites in the genome [13], further confirmed our observation, although two neighboring guanines (G) upstream of the dominant transcription start site were also predicted to be potential transcription start points, but with less probability. These analyses supported the classification of *KIAA1199* as a single dominant peak promoter according to the Carninci promoter classification method in which four types of promoters are given [14]. In addition, the transcription start site of *KIAA1199* is conserved between human and mouse.

Another common structure in the *KIAA1199* 5′-flanking region is a GC-box that is located in the −248/−243 region of the *KIAA1199* promoter. Since an effective GC-box is generally found in the region 70–80 bp upstream from the transcription state site [19], we hypothesized that the GC-box of *KIAA1199* may not be required for regulation of *KIAA1199* promoter activity. This hypothesis was supported by our deletion mutant study. Methylation of cytosine residues is an important feature of GC-boxes in regards to regulation of gene expression [20]. Although this GC-box containing region is predicted to be within the CpG island, we did not observe any methylation of cytosine in the GC-box. In addition, deletion of the GC-box in the *KIAA1199* promoter did not change reporter gene activity, further suggesting that methylation at the GC-box is not involved in regulation of *KIAA1199* expression. Since it has been shown that the Sp-1 transcription factor binds to GC-boxes [21] to regulate gene expression, further studies are needed to fully understand the function of GC-box. Similarly, we found that Twist-1, a transcription factor involved in EMT conversion in epithelial cancer cells [16], was not required for regulation of *KIAA1199*, although putative *cis*-elements were present.

Through a series of deletions of the 5′-flanking region of *KIAA1199*, we revealed three important DNA fragments in the regulation of *KIAA1199* promoter activity: 1) an inhibitory region between −2341 and −1425, 2) a distal activator region between −1425 and −1135, and 3) a proximal activator region between −125 and +27. We observed the strongest promoter activity in the 1.6 kb fragment ranging from −1425 to +254. This promoter activity was decreased by more than 50% when 2.3 and 3.3 kb *KIAA1199* 5′-flanking regions were examined in COS-1 cells as well as in MCF-7 and MDA-MB-231 breast cancer cells, suggesting the presence of negative regulatory *cis*-acting elements in the upstream region between −1425 and −2341. Bioinformatics analysis revealed multiple negative glucocorticoid response elements (nGRE) in the −2204/−2190, −2189/−2175 and −1780/−1766 regions. nGRE has been demonstrated to suppress the promoter activity in the presence of glucocorticoids [22], suggesting that *KIAA1199* might be under the negative control of a glucocorticoids-nGRE pathway. This possibility is currently being examined.

Our study demonstrated a pivotal role of AP-1 in controlling *KIAA1199* expression since the activity of the pro-1.4 kb *KIAA1199* promoter was repressed by more than 80% by either deleting or swapping the AP-1 consensus sequence. AP-1 proteins are primarily considered to be oncogenic and have been reported to upregulate genes involved in cancer dissemination,for example matrix metalloproteinase (MMP) −2, and −9 [23]. EMSA data in this study indicated that probes containing the AP-1 binding sequence were bound to AP-1 proteins, possibly Fos/Jun heterodimers. Two shifted bands (protein-DNA complexes) in EMSA were detected in the presence of a double stranded biotinylated-oligonucleotide containing the DNA binding sequence for AP*-*1. However, Supershift EMSA in the presence of a c-Jun-specific antibody created a supershift band of Shift-2 band, with no change with the Shift-1 band, suggesting that the Shift-2 band is the AP-1-DNA complex. This conclusion is further supported by the observation that the Shift-1 band was not changed when a probe with a mutant AP-1 consensus sequence was used (Fig. 5). Therefore, we conclude that AP-1 specifically binds to the AP-1 consensus sequence. Since the 50-bp oligonucleotide probe contains the TATA-box sequence that has been shown to bind to TATA-box binding protein (TBP) [24], we assume that the Shift-1 band may result from the binding between TBP and the probe based on the fact that the molecular weight of TBP is less than Fos/Jun heterodimers.

Although four putative NF-κB *cis*-elements were present, we demonstrated that the functional *cis*-element of NF-κB is located in the distal part of the promoter, as determined by mutagenesis study, EMSA, and ChIP analyses. Interestingly, only this NF-κB *cis*-element is highly conserved between human and mouse genomes. Despite the fact that it is less common for a regulatory element within a distal part of a promoter to have a functional role, there is evidence demonstrating the localization of functional elements in distal parts of promoters [25]. Identification of the NF-κB site as an activator element in the *KIAA1199* promoter is significant because NF-κB is essential for epithelial cancer EMT and metastasis [15]. Of interest, a synergistic effect of NF-κB and AP-1 in tumorigenesis has been documented in colon and breast cancers [26]. Accordingly, understanding the role of the AP-1 and NF-κB *cis*-elements in the regulation of *KIAA1199* may help in the development of strategies aimed at preventing cancer dissemination.

In addition to genetic regulation, DNA methylation is also involved in the regulation of *KIAA1199* expression. Although the predicted *KIAA1199* CpG island starts in the promoter and extends into the first intron, our experimental data demonstrated that the effective region of the CpG island is in the first intron area of the predicted *KIAA1199* CpG island (−444 to +1509). We observed methylation of the CpG island within the first intron of *KIAA1199* in non- or minimally-*KIAA1199* expressing cell lines. However, this region is demethylated in aggressive human breast cancer cell lines and in cancer cells isolated from human breast cancer specimens. Hypermethylation of specific CpG sites within the first exon, intron, or downstream region of promoters has previously been reported to suppress gene expression [27]. In general, 40–50% of human genes have predicted CpG islands around their promoter region, but most of them are not methylated [28], for example there is no DNA methylation in housekeeping genes even when predicted CpG islands are present in these genes. It is well known that DNA methylation contributes to tissue specific expression [29]. Although methylation of *KIAA1199* has been reported in normal colon mucosa and demethylation in human colon carcinoma specimens by a high-throughput analysis [6], there is no detailed analysis of the quantification of methylation at individual CG pairs for *KIAA1199*. Our study demonstrated for the first time mapping of specific methylated cytosines in the CpG island of *KIAA1199*.

It is generally accepted that two mechanisms account for transcriptional repression via DNA methylation. In addition to directly inhibiting the binding of transcription factors, such as AP-2, c-Myc, E2F, and NF-κB to their binding sites within promoter [30], methylation of CpG islands in non-*cis*-element containing regions, for example extrons or introns, could present a significant obstacle to the processive transcription complexes by altering chromatin structure, thus inhibiting transcription elongation [31]. Since there are no transcription factor binding sites in the second region of the *KIAA1199* CpG island, we assume that prevention of transcription elongation occurs in non-*KIAA1199* expressing cells resulting in little to no expression of *KIAA1199*. In support of this notion, we have identified increased H3K27 trimethylation on histone tails of nucleosomes at the second region of the CpG island of *KIAA1199* in human umbilical vein endothelial cells (HUVEC) and MCF-7 cells, both cell lines expressing low levels of *KIAA1199*, as compared to H3K27 trimethylation in MDA-MB-231 cells (data not shown). In addition, MDA-MB-231 cells have high H3K4 trimethylation in the second-subregion supporting the higher expression of *KIAA1199* in this cell type. This is possibly due to the fact that methylation of H3K27 potentially leads to more condensed chromatin creating an obstacle that prevents transcription elongation. Additional experiments are necessary to further address these possibilities.

Although DNA mehtylation events in cancer are primarily considered in the inactivation of tumor suppressor genes [10], mounting evidence has demonstrated that demethylation occurs in cancer progression and results in upregulation of cancer-related genes [32]–[39]. However, whether methylation directly elicits gene inactivation or is an outcome of gene silencing remains to be determined. Nevertheless, detection of aberrant DNA methylation is considered a promising diagnostic tool in cancer [40]. Our studies identifying a correlation between DNA methylation and human breast cancers provide a novel alternative approach for diagnosis of human breast cancer.

## Supporting Information

Figure S1 **Overexpression of c-Jun (AP-1) and p65 (NFκB), but not Twist-1, increase expression of** ***KIAA1199*** **.** **A)** Western blotting analysis of cell lysates from MDA-MB 231 cells transfected with Twist-1, c-Jun (AP-1) or p65 (NFκB) cDNAs. β-actin was used as a loading control. **B)** Total RNA from transfected MDA-MB 231 cells was analyzed via real-time RT-PCR using primers specific for *KIAA1199*. The expression level of *KIAA1199* was normalized using HPRT-1 and GAPDH housekeeping genes. Each bar represents the mean ± S.E (*<0.05).
(TIF)

Figure S2 **Silencing of c-Jun decreases** ***KIAA1199*** **mRNA level.** A) Total RNA from MDA-MB-231 cells expressing either GFP (control) shRNA or shRNA against c-Jun was analyzed via real time RT-PCR using primers specific for *KIAA1199*. The expression level was normalized using HPRT-1and GAPDH housekeeping genes. Each bar represents the mean ± S.E (*<0.05).
(TIF)

Figure S3 **Silencing of c-Jun or p65 results in decreased** ***KIAA1199*** **promoter activity. A)** COS-1 cells were infected with retrovirus encoding GFP shRNA (control), c-Jun shRNA and stable cells were pooled following puromycin selection. Total RNA was analyzed by real time RT-PCR to verify knockdown. Expression levels were normalized using HPRT-1 and GAPDH housekeeping genes. Each bar represents the mean ± S.E. *Left panel*: Reduced protein expression level of c-Jun was validated by western blotting. β-actin was used as a loading control. B) p65 was also silenced in COS-1 cells with similar approach like c-Jun. **C)** COS-1 cells expressing indicated shRNA were transfected with the pro-1.4 kb *KIAA1199* promoter luciferase reporter cDNA along with Renilla cDNA. Promoter activity was analyzed using the Dual-Glo Luciferase assay system. The increased *KIAA1199* promoter activity as compared to pGL3 basic luciferase reporter was abrogated upon silencing of either c-Jun or p65.
(TIF)

Figure S4 **Verification of methylation specific PCR.** The U pair (unmethylated specific primer) and the M pair (methylated specific primer) primers were validated by using both control unmethylated human genomic DNA and methylated human genomic DNA (Sss.i treated).
(TIF)

Figure S5 **Laser Capture Microdissection (LCM) of tumor and normal cells from breast tissue specimens. A)** Normal breast epithelial cells and malignant cancer cells were identified following Hemotoxylin-Eosin (H&E) staining of tissue sections and then collected via LCM using the Lieca Laser Microscope. **B)** The cancer cells from in-situ breast cancer specimens were outlined by red lines and numbered with green boxes. The laser captured cells were then used for mRNA analysis and methylation profiling of the CpG island within *KIAA1199*. **C)** A representative image after laser microdissection.
(TIF)

Table S1 **Primer sequences for promoter constructs.**
(DOCX)

Table S2 **Oligonucleotides in EMSA studies.**
(DOCX)

Table S3 **Primers for ChIP assays.**
(DOCX)

Table S4 **MSP, UMP and BSP primers.**
(DOCX)

Table S5 **Oligonucleotides in silencing studies.**
(DOCX)
