# Supplementary figures and images for: Transcriptional and Epigenetic Regulation of KIAA1199 Gene Expression in Human Breast Cancer
Source: PLoS One. 2012 Sep 6;7(9):e44661. doi: 10.1371/journal.pone.0044661 (PMC3435267; doi:10.1371/journal.pone.0044661)

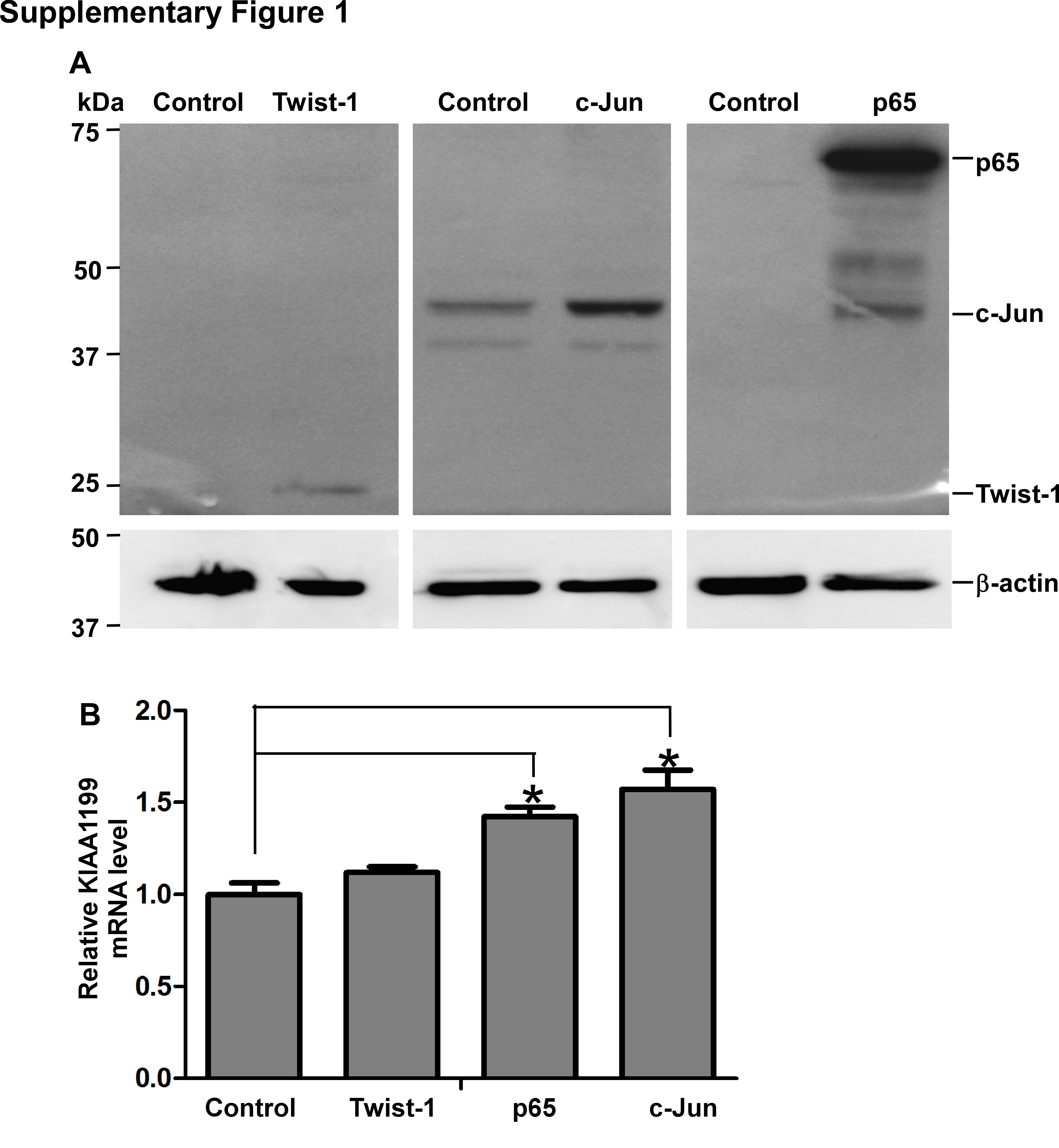

Supplement: Figure S1 — Overexpression of c-Jun (AP-1) and p65 (NFκB), but not Twist-1, increase expression of KIAA1199 . A) Western blotting analysis of cell lysates from MDA-MB 231 cells transfected with Twist-1, c-Jun (AP-1) or p65 (NFκB) cDNAs. β-actin was used as a loading control. B) Total RNA from transfected MDA-MB 231 cells was analyzed via real-time RT-PCR using primers specific for KIAA1199. The expression level of KIAA1199 was normalized using HPRT-1 and GAPDH housekeeping genes. Each bar represents the mean ± S.E (*<0.05). (TIF) [file pone.0044661.s001.tif]

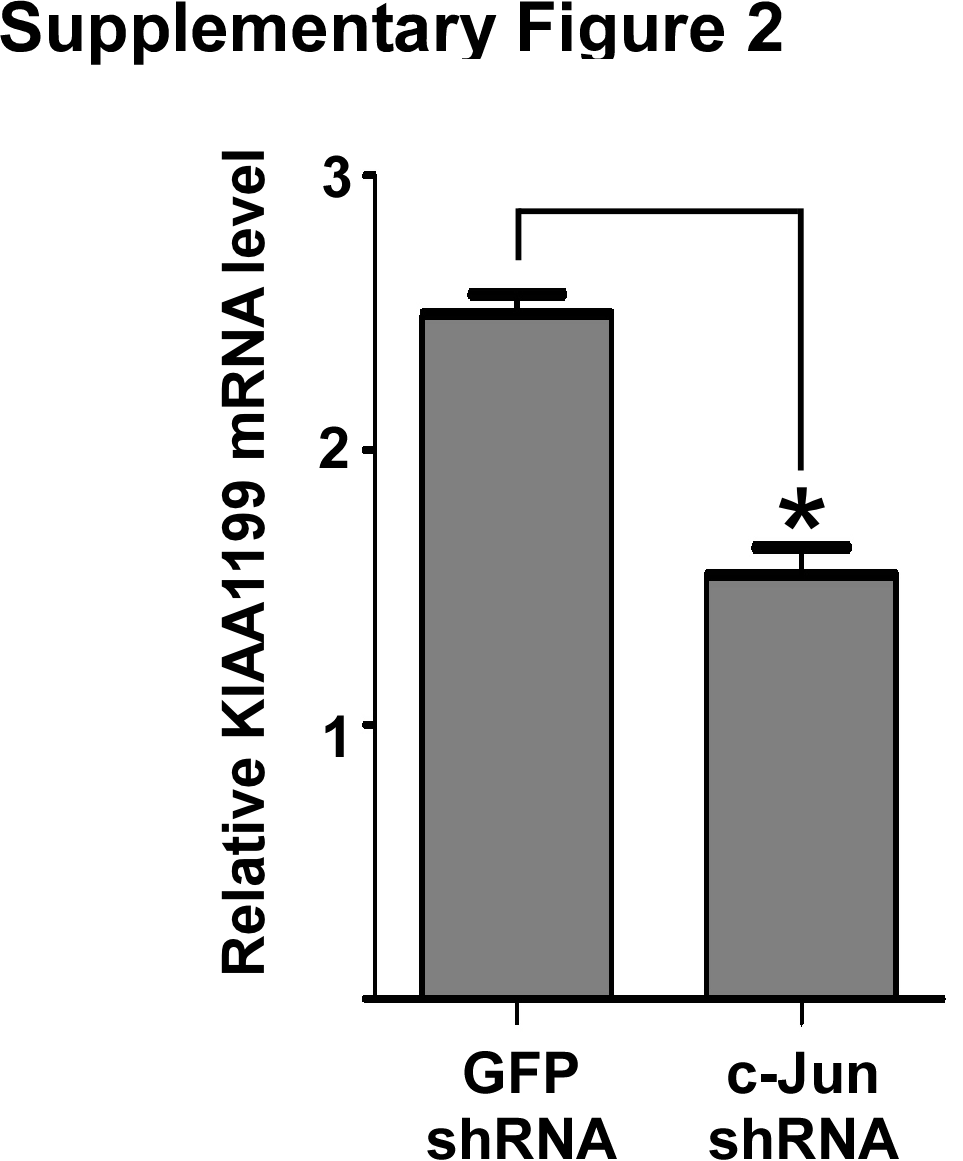

Supplement: Figure S2 — Silencing of c-Jun decreases KIAA1199 mRNA level. A) Total RNA from MDA-MB-231 cells expressing either GFP (control) shRNA or shRNA against c-Jun was analyzed via real time RT-PCR using primers specific for KIAA1199. The expression level was normalized using HPRT-1and GAPDH housekeeping genes. Each bar represents the mean ± S.E (*<0.05). (TIF) [file pone.0044661.s002.tif]

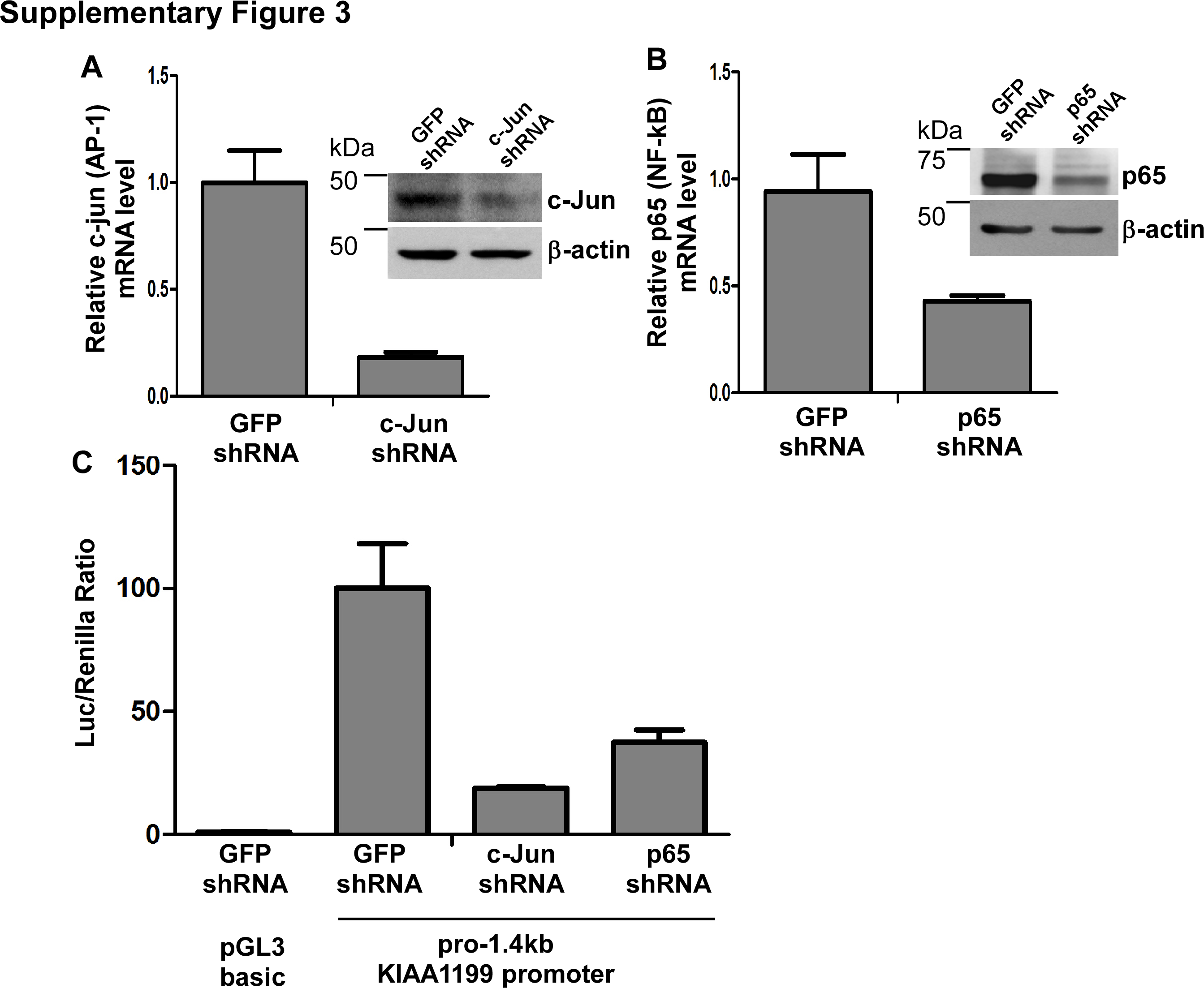

Supplement: Figure S3 — Silencing of c-Jun or p65 results in decreased KIAA1199 promoter activity. A) COS-1 cells were infected with retrovirus encoding GFP shRNA (control), c-Jun shRNA and stable cells were pooled following puromycin selection. Total RNA was analyzed by real time RT-PCR to verify knockdown. Expression levels were normalized using HPRT-1 and GAPDH housekeeping genes. Each bar represents the mean ± S.E. Left panel: Reduced protein expression level of c-Jun was validated by western blotting. β-actin was used as a loading control. B) p65 was also silenced in COS-1 cells with similar approach like c-Jun. C) COS-1 cells expressing indicated shRNA were transfected with the pro-1.4 kb KIAA1199 promoter luciferase reporter cDNA along with Renilla cDNA. Promoter activity was analyzed using the Dual-Glo Luciferase assay system. The increased KIAA1199 promoter activity as compared to pGL3 basic luciferase reporter was abrogated upon silencing of either c-Jun or p65. (TIF) [file pone.0044661.s003.tif]

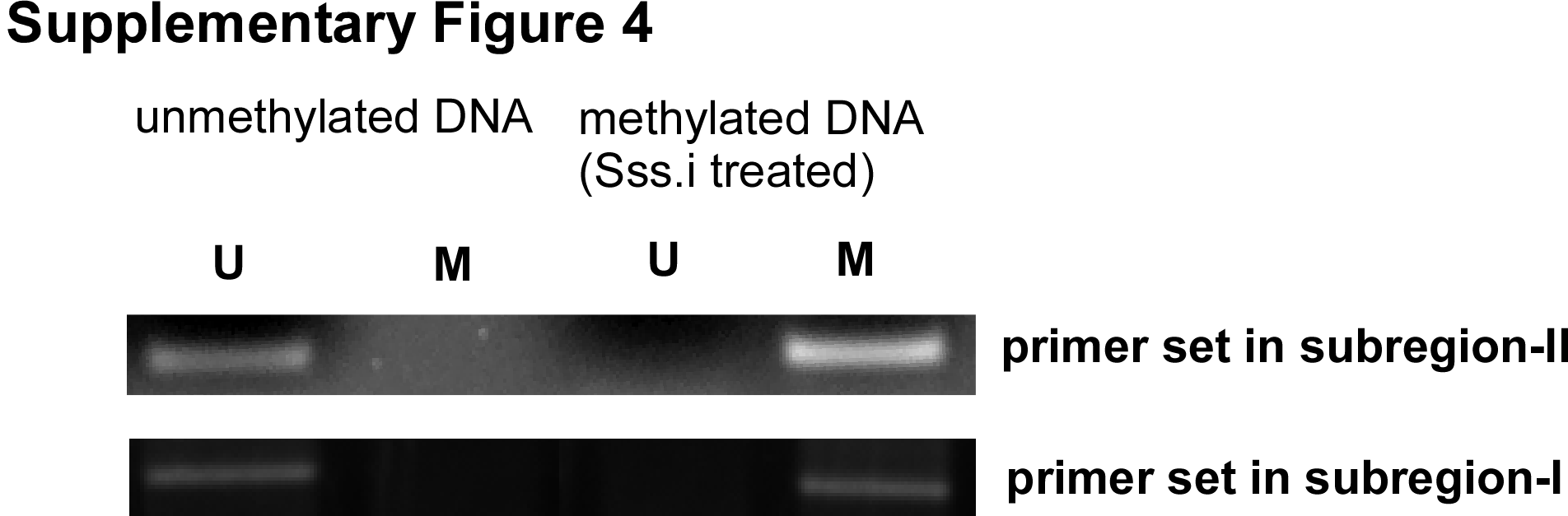

Supplement: Figure S4 — Verification of methylation specific PCR. The U pair (unmethylated specific primer) and the M pair (methylated specific primer) primers were validated by using both control unmethylated human genomic DNA and methylated human genomic DNA (Sss.i treated). (TIF) [file pone.0044661.s004.tif]

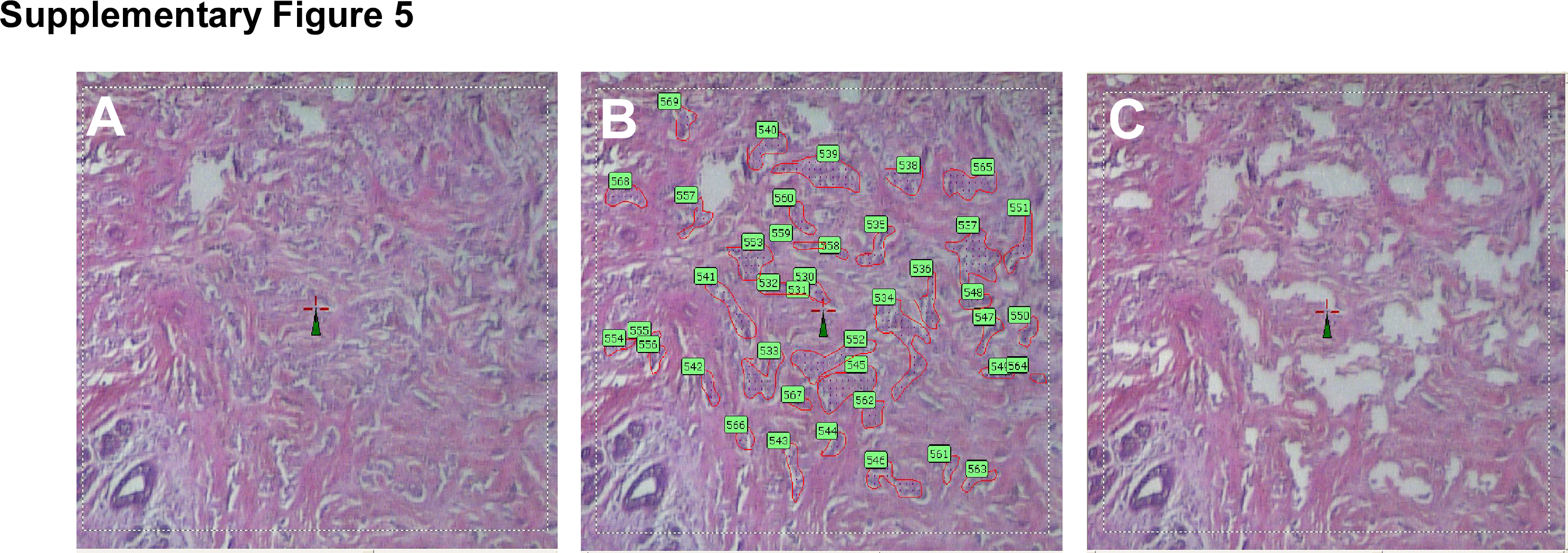

Supplement: Figure S5 — Laser Capture Microdissection (LCM) of tumor and normal cells from breast tissue specimens. A) Normal breast epithelial cells and malignant cancer cells were identified following Hemotoxylin-Eosin (H&E) staining of tissue sections and then collected via LCM using the Lieca Laser Microscope. B) The cancer cells from in-situ breast cancer specimens were outlined by red lines and numbered with green boxes. The laser captured cells were then used for mRNA analysis and methylation profiling of the CpG island within KIAA1199. C) A representative image after laser microdissection. (TIF) [file pone.0044661.s005.tif]
