# Supplementary material for: Transcriptional and Epigenetic Regulation of KIAA1199 Gene Expression in Human Breast Cancer
Source: PLoS One. 2012 Sep 6;7(9):e44661. doi: 10.1371/journal.pone.0044661 (PMC3435267; doi:10.1371/journal.pone.0044661)
Supplement: Table S1 — Primer sequences for promoter constructs. (DOCX) [file pone.0044661.s006.docx]

**Table S1**

Primer sequences for promoter constructs. KpnI one site in forward primers and BglII site in reverse primers were underlined.

| primer name | primer sequence |
| --- | --- |
| Pro-3.3 kb | 5’ATGGTACCCATGGAAAAATGCTTGGCTA |
| Pro-2.3 kb | 5’ATGGTACCCAGGCCAATCTACTGCACAA |
| Pro-1.4 kb | 5’ATGGTACCCAAAGAAGGGCTGAGAATCC |
| Pro-1.135 | 5’ATGGTACCTAGGAGGCCTCTGCTAACCA |
| Pro-0.913 | 5’ATGGTACCATGCCCTAGGGATGTGTTGT |
| Pro-0.583 | 5’ATGGTACCTTCAAACCAAGGTGCCTTTC |
| Pro-0.217 | 5’ATGGTACCGCTCAGGTCTGCGTCTCC |
| Pro-0.125 | 5’ATGGTACCGCGTGGAGGGAAGTTTCAT |
| Pro+0.027 | 5’ATGGTACCGCGGTGCTATCGGACAGAG |
| Reverse | 5’ATAGATCTGCCCTCTTACCTCTGGGTCT |
