# Supplementary material for: Transcriptional and Epigenetic Regulation of KIAA1199 Gene Expression in Human Breast Cancer
Source: PLoS One. 2012 Sep 6;7(9):e44661. doi: 10.1371/journal.pone.0044661 (PMC3435267; doi:10.1371/journal.pone.0044661)
Supplement: Table S2 — Oligonucleotides in EMSA studies. (DOCX) [file pone.0044661.s007.docx]

**Table S2**

EMSA probes. Substituted nucleotides are shown with small letters. Nucleotides are underlined if Consensus Index score (Ci-value) >60 to be a *cis*-acting site.

| primer name | primer sequence |
| --- | --- |
| AP-1 sense | 5’CGTCAGGGGCTCCTCGGGGGAGGAGTCAGTAAAAGTGGCTATAAAAGCGG |
| AP-1 antisense | 5’CCGCTTTTATAGCCACTTTTACTGACTCCTCCCCCGAGGAGCCCCTGACG |
| AP-1 mutant sense | 5’GTCAGGGGCTCCTCGGGGGAGagacCAGTAAAAGTGGCTATAAAAGC |
| AP-1 mutant antisense | 5’GCTTTTATAGCCACTTTTACTGgtctCTCCCCCGAGGAGCCCCTGAC |
| NFκB sense | 5’AGAGGCCTAGAGGTGGATTGCAGGACTTCCCAGTTTAACCAGGTGACAAA |
| NFκB antisense | 5’TTTGTCACCTGGTTAAACTGGGAAGTCCTGCAATCCACCTCTAGGCCTCT |
| NFκB mutant sense | 5'CTGGAAGAGGCCTAGAGGTGGATTGCgaagCcctCCAGTTTAACCAGGTGACAAAGGCTT |
| NFκB mutant antisense | 5'AAGCCTTTGTCACCTGGTTAAACTGGaggGcttcGCAATCCACCTCTAGGCCTCTTCCAG |
