# Supplementary material for: Transcriptional and Epigenetic Regulation of KIAA1199 Gene Expression in Human Breast Cancer
Source: PLoS One. 2012 Sep 6;7(9):e44661. doi: 10.1371/journal.pone.0044661 (PMC3435267; doi:10.1371/journal.pone.0044661)
Supplement: Table S3 — Primers for ChIP assays. (DOCX) [file pone.0044661.s008.docx]

**Table S3**

Primers used during ChIP assays.

| primer name | primer sequence |
| --- | --- |
| AP-1 A-For | 5’GCGTGGAGGGAAGTTTCAT |
| AP-1 A-Rev | 5’AGGCCGCTTTTATAGCCACT |
| AP-1 B-For | 5’TGGAAGAAGGTCTGGTGGTC |
| AP-1 B-Rev | 5’CTCTCATGAGCACACGCATC |
| NFκB A-For | 5’GGATCAAGTTCCGCTTTCTG |
| NFκB A-Rev | 5’TGATCACATCTGGGCTGAAG |
| NFκB B-For | 5’CGCAGACCTTTTCTCCTGTC |
| NFκB B-Rev | 5’CTCTGGGAGACGCAGACCT |
| NFκB C-For | 5’TGGAAGAAGGTCTGGTGGTC |
| NFκB C-Rev | 5’CTCTCATGAGCACACGCATC |
