# Supplementary material for: Transcriptional and Epigenetic Regulation of KIAA1199 Gene Expression in Human Breast Cancer
Source: PLoS One. 2012 Sep 6;7(9):e44661. doi: 10.1371/journal.pone.0044661 (PMC3435267; doi:10.1371/journal.pone.0044661)
Supplement: Table S4 — MSP, UMP and BSP primers. (DOCX) [file pone.0044661.s009.docx]

**Table S4**

MSP, UMP and BSP primers

| primer name | primer sequence |
| --- | --- |
| MSP1-For | 5’TTAGCGTTTAAGTAGAGTTTAGCGC |
| MSP1-Rev | 5’CCGAAACGTAAACAAACGAA |
| UMP1-For | 5’AGTGTTTAAGTAGAGTTTAGTGTGG |
| UMP1-Rev | 5’AGTGTTTAAGTAGAGTTTAGTGTGG |
| MSP2-For | 5’GTTCGGGAGAGTGTTTCGTC |
| MSP2-Rev | 5’GCGTCGCTAAATACCTAACG |
| UMP2-For | 5’GTTTGGGAGAGTGTTTTGTTGA |
| UMP2-Rev | 5’AACACATCACTAAATACCTAACACT |
| BSP1-For | 5’GGGTTATTTTTTTGTTGTTGAGTT |
| BSP1-Rev | 5’bio-TTATAACCACTTTTACTAACTCCT |
| seq1.1 | 5’TTGAGATTTAGTTTGGGA |
| BSP2-For | 5’TTAGAGGGTATTTAGAGGGGGTAGA |
| BSP2-Rev | 5’bio-CCTTAAAATCCATTCCAAAATATCA |
| seq2.1 | 5’TTTTTTTTTTTGAATGGT |
| seq2.2 | 5’AGTATTATAAGGGTTAGGTT |
| seq2.3 | 5’GATTGGGAGTTAGTTTA |
| seq2.4 | 5’ACATTACAATTACCTCAACT |
| seq2.7 | 5’GATGTTTAGATTGAGATTTT |
| seq2.8 | 5’TGGTTTTTGGTAAAGGA |
| seq2.9 | 5’GGTAGAGAGTTTTGGTAGAA |
| BSP2-For | 5’bio-TTAGAGGGTATTTAGAGGGGGTAGA |
| BSP2-Rev | 5’CCTTAAAATCCATTCCAAAATATCA |
| seq2.5 | 5’TTTTTTTTTTTAGTTTTTTT |
| seq2.6 | 5’AAAAAAACTAAAAAAAAA |
