# Supplementary material for: Transcriptional and Epigenetic Regulation of KIAA1199 Gene Expression in Human Breast Cancer
Source: PLoS One. 2012 Sep 6;7(9):e44661. doi: 10.1371/journal.pone.0044661 (PMC3435267; doi:10.1371/journal.pone.0044661)
Supplement: Table S5 — Oligonucleotides in silencing studies. (DOCX) [file pone.0044661.s010.docx]

**Table S5**

Oligonucleotide sequences for silencing of c-Jun and p65 in pSiren RetroQ system. Nucleotides matched to the mRNA of target gene were underlined.

| c-Jun sense | 5’GATCCAAGAACGTGACAGATGAGCAGGCTTCCTGTCACCTGCTCATCTGTCACGTTCTTTTTTTTG |
| --- | --- |
| c-Jun antisense | 5’AATTCAAAAAAAAGAACGTGACAGATGAGCAGGTGACAGGAAGCCTGCTCATCTGTCACGTTCTTG |
| p65 sense | 5’GATCCCCATCAACTATGATGAGTTTCGCTTCCTGTCACGAAACTCATCATAGTTGATGGTTTTTTG |
| p65 antisense | 5’AATTCAAAAAACCATCAACTATGATGAGTTTCGTGACAGGAAGCGAAACTCATCATAGTTGATGGG |
